# Adaptive Plasticity in the Healthy Language Network: Implications for Language Recovery after Stroke

**DOI:** 10.1155/2016/9674790

**Published:** 2016-10-18

**Authors:** Gesa Hartwigsen

**Affiliations:** Department of Neuropsychology, Max Planck Institute for Human Cognitive and Brain Sciences, Leipzig, Germany

## Abstract

Across the last three decades, the application of noninvasive brain stimulation (NIBS) has substantially increased the current knowledge of the brain's potential to undergo rapid short-term reorganization on the systems level. A large number of studies applied transcranial magnetic stimulation (TMS) and transcranial direct current stimulation (tDCS) in the healthy brain to probe the functional relevance and interaction of specific areas for different cognitive processes. NIBS is also increasingly being used to induce adaptive plasticity in motor and cognitive networks and shape cognitive functions. Recently, NIBS has been combined with electrophysiological techniques to modulate neural oscillations of specific cortical networks. In this review, we will discuss recent advances in the use of NIBS to modulate neural activity and effective connectivity in the healthy language network, with a special focus on the combination of NIBS and neuroimaging or electrophysiological approaches. Moreover, we outline how these results can be transferred to the lesioned brain to unravel the dynamics of reorganization processes in poststroke aphasia. We conclude with a critical discussion on the potential of NIBS to facilitate language recovery after stroke and propose a phase-specific model for the application of NIBS in language rehabilitation.

## 1. An Introduction to the Study of Language Networks

The ability to associate sound patterns with meaningful concepts and articulate one's thoughts is a core feature of human communication. During successful language processing, rapid analysis of sound, meaning, and structure of spoken or written words is required. Since the days of Broca and Wernicke in the second half of the 19th century, researchers aimed at identifying key networks for language comprehension and production in the human brain. The first functional-anatomical models of language were solely based on the observation of behavioural deficits in patients with brain lesions and relied on postmortem analyses of damaged brain areas (e.g., [1, 2]; see [3] for review). The advent of modern electrophysiological and neuroimaging techniques like electroencephalography (EEG), magnetoencephalography (MEG), positron emission tomography (PET), and functional magnetic resonance imaging (fMRI) in the late 20th century allowed for the direct correlation between mental operations and neural activity in the healthy human brain [4]. These approaches were complemented by the application of noninvasive brain stimulation (NIBS) that enables the researcher to probe the causal relevance of task-specific neural activity for different motor or cognitive functions. Moreover, when applied in a plasticity-inducing fashion, NIBS further allows for the investigation of rapid short-term reorganization on the systems level.

The capacity of the human brain to flexibly change the functional weight within a network is a core feature of adaptive reorganization and compensation after brain lesions. A profound understanding of language organisation and the brain's general potential for adaptive plasticity is mandatory for the interpretation of reorganization processes in the lesioned brain and might ultimately prove useful to optimize treatment strategies for language rehabilitation in patients with poststroke aphasia.

In this review, we will discuss how different NIBS approaches can be used to investigate adaptive plasticity in the healthy and lesioned language network and elucidate the potential of such approaches to enhance recovery of language function after stroke-induced brain lesions. First, some basic mechanism of transcranial magnetic stimulation (TMS) and transcranial direct current stimulation (tDCS) will be discussed. We will also introduce more recent NIBS approaches like transcranial alternating current stimulation (tACS) and transcranial random noise stimulation (tRNS). With respect to the application of these techniques, the emerging field of computational neurostimulation [5] might substantially advance the experimental and clinical use of NIBS by establishing quantitative models that link stimulation dose to behavioural and clinical outcomes and provide insight into the physiological underpinnings of the stimulation effects [6].

In the second part of the review, we will focus on the combination of NIBS and neuroimaging or electrophysiological techniques in the healthy language network. Employing multimethod approaches allows for a comprehensive mapping of stimulation-induced effects on neural activity and connectivity locally and in distant connected network regions and provides insight into the adaptive short- and long-term effects induced by NIBS.

## 2. An Introduction to Noninvasive Brain Stimulation

The application of electrical currents to stimulate body parts dates back to Galvani (1737–1798) who pioneered the field of bioelectromagnetics with his discovery that the muscles of a dead frog's legs twitched when struck by an electrical spark, an observation that he referred to as “animal electricity” [7]. About 100 years later, Fritsch and Hitzig [8] demonstrated that electrical stimulation of different cortical areas caused involuntary muscular contractions of various body parts in dogs, thereby dethroning the doctrine that the brain was electrically inexcitable.

### 2.1. Transcranial Magnetic Stimulation (TMS)

It took another 100 years after Fritsch's and Hitzig's discoveries until transcranial magnetic stimulation (TMS) was introduced as a noninvasive technique for electrical stimulation of the human cortex by Barker and colleagues in 1985 [9]. Strikingly, the principles of electromagnetism that underlie TMS were well known more than a century before its introduction, and the failure to develop TMS sooner was due to a lack of the necessary high-power electronics [10].

TMS is based on the principles of electromagnetic induction. A brief electric current produces a strong time-varying magnetic field in the TMS coil, and the time-varying magnetic field penetrates the scalp without attenuation to induce a flow of electric current in the stimulated tissue [11]. A single TMS pulse thereby causes electro-magneto-electric stimulation of neuronal axons, particularly in superficial regions of the cerebral cortex that can temporarily excite or inhibit the stimulated area [12]. A large number of previous studies have elucidated the physiological mechanisms of TMS in the human motor system (e.g., [11–14]). When applied over the primary motor cortex, TMS can depolarize corticospinal tract neurons and evoke contralateral hand muscle movements. The size of these motor evoked potentials reflects the excitability of the corticospinal system [11]. However, the physiological mechanisms of the TMS-induced neuronal excitation at the cellular level remain largely unclear. For instance, tissue resistivity and cerebrospinal fluid likely influence current flow, electric field direction, and magnitude [15, 16] and it remains illusive how the cellular and gyral shapes or grey matter boundaries influence stimulation effects [17].

An increasing number of studies used biophysical modelling and simulation of the electric field distribution induced by TMS or transcranial direct current stimulation (tDCS) to provide estimations of the spatial stimulation patterns induced by these techniques (see [18]). The results from these modelling studies might inform future applications of NIBS and thereby increase the spatial specificity of these methods.

The effects of TMS on cognitive functions can be probed on the behavioural level by changes in response speed or accuracy or alterations in neural responses mapped by M/EEG, PET, and fMRI. Behavioural TMS studies usually employ TMS bursts to characterize the functional relevance of task-specific activity patterns observed in neuroimaging studies, following the assumption that if a certain area is critical to a given task, then transient disruption of this region with TMS should impair task processing, which should lead to a measurable change in the dependent variable of interest [14, 19].

#### 2.1.1. Complementary TMS Approaches: Online versus Offline TMS

Nowadays, a large number of different TMS protocols have become available, ranging from the application of single or double pulses to short or long bursts of repetitive TMS (rTMS) with different frequencies [20]. TMS is usually applied either before a task (i.e., offline) or during a task (i.e., online).

*Online TMS* is particularly suited to directly interfere with ongoing task processing and provide causal structure-function relationships [21–23]. The acute, transient effect of online TMS leaves the brain no time for functional reorganization and is thus not confounded by chronic processes of functional recovery [23, 24]. This is an important advantage of studying TMS induced perturbation relative to the investigation of structural brain lesions in clinical settings. In contrast to structural lesions that seldom conform to functionally homogenous neuroanatomical subsystems, TMS is more focal (average resolution of about 1–1.5 cm) and allows for the perturbation of different subregions within a larger area of interest [25]. The majority of online TMS studies in the language domain applied either single pulses or high-frequency 10 Hz bursts over one cortical area, although some designs also included double and triple pulses at higher frequencies, low-frequency bursts, or multifocal TMS over more than one region [26]. It remains to be determined why 10 Hz is efficient in disrupting language performance. One might speculate that this protocol modulates ongoing language-related oscillatory frequencies in the stimulated area, probably by inducing alpha entrainment.

*Offline TMS*, on the other hand, can be used to study processes of adaptive plasticity on the systems level [27, 28]. Offline TMS usually refers to the application of repetitive TMS (rTMS) that can suppress task-related activity for an extended time period (usually about 30–45 minutes). The offline approach bears some analogies to acute stroke, because it may give rise to an acute adaptive reorganization within the nonstimulated functional nodes of the network to compensate for the TMS-induced suppression of neural activity in those components of the network that have been perturbed with TMS [28, 29]. Notably, some rTMS protocols like intermittent theta-burst stimulation can also facilitate motor cortical excitability [30] and probably also some cognitive processes in the healthy brain, including language [31] and working memory [32]. Such a protocol might thus prove useful to promote language recovery after stroke [33].

The neurophysiological mechanisms of plasticity-inducing rTMS protocols are poorly understood. A common assumption is that rTMS influences neural excitability by long-term potentiation- and depression- (LTP- and LTD-) like effects of synaptic processes [17, 34]. Such after effects of TMS can be mapped with neuroimaging and electrophysiological techniques and were reported to modulate behavioural performance in different language tasks (e.g., [35–37]; see below for details).

#### 2.1.2. Stimulation Parameters for TMS Studies: Control Conditions and Intensity

A critical issue for all TMS studies is the appropriate choice of a control condition. Many studies rely on placebo stimulation (referred to as sham TMS) to control for unspecific side effects, such as the clicking sounds that are produced when the coil is discharged. However, placebo stimulation is usually easy to identify as ineffective for the participant, especially when compared to stimulation of core language areas, where high stimulation intensities can induce muscle twitches and discomfort caused by direct stimulation of nerves. Depending on the network under investigation, a neighbouring region that is not expected to contribute to the task of interest or a contralateral homologous region could be chosen as active control region.

Another important issue that needs to be taken into account when applying TMS is that the induced electric field decreases rapidly with increasing distance from the coil. Hence, only a few regions on the cortical surface can be directly stimulated with TMS while deep brain structures might only be indirectly targeted. The stimulation intensity itself also influences the effectiveness of the applied protocol. For instance, Brückner et al. [38] demonstrated that continuous theta-burst stimulation (cTBS) over the prefrontal cortex only impaired lexical decisions when applied at 90% of the individual active motor threshold, but not with the “standard” intensity of 80% of the active motor threshold [30].

### 2.2. Transcranial Direct Current Stimulation (tDCS)

tDCS has been used to study plasticity in animals for a long time before it was reintroduced for application in the human brain by Nitsche and Paulus in 2000 [39]. Animal studies revealed that weak polarizing currents applied to the brain surface could produce lasting changes in cortical-evoked potentials and influence the activity of individual cortical neurons [40–42]. During tDCS, weak direct electrical currents of 1-2 mA are applied continuously to the scalp between two large sponge electrodes for usually up to 20–30 minutes [43].

Although the physiological effects of tDCS are not fully understood, it is argued that surface-anodal polarization of the cortex with the anode near the dendritic poles of radially oriented neurons increases the firing rates of spontaneously active cells, while cathodal polarization has the opposite effect [39]. Importantly, tDCS does not cause spontaneous firing but is thought to primarily work via a passive change in the resting membrane potential [44, 45] through voltage-gate ion channels [46, 47]. As the electric field diffuses rapidly in the head, the physiological action of the current is presumably near the surface [48]. Physical models suggest that approximately half of the applied current is shunted through the scalp [49] and another significant amount through the cerebrospinal fluid [50]. In this context, it should be noted that very recent (yet unpublished) data obtained from electrode recordings in a human cadaver by Buzsaki and colleagues suggested that up to 90% of the current had been redirected by the skin (see comment by Underwood [51]), questioning the effectiveness of tDCS and related techniques to stimulate brain tissue. However, it remains unclear whether such postmortem results translate to the conductivity of the living human brain.

The focality of tDCS is not known, but modelling studies indicate that a large area under the electrode is polarized [49]. It is generally assumed that the strongest tDCS effect should occur at the stimulated area under the electrode [52], but functional effects also engage distant neural networks [53], and the position of the second electrode probably affects the effects under the first one [54]. Common montages place both electrodes on the head in a bipolar arrangement, although in theory, the reference electrode can be placed anywhere on the body to ensure that it exerts no physiological effects of its own [6]. Given the overall low focality of tDCS, a direct structure-function relationship is hard to establish, especially with respect to the induced behavioral changes [55].

One important feature of tDCS is the ability to modulate cortical excitability for longer time periods [39]. For instance, plasticity related after effects of tDCS on the behavioural level were reported up to 6–12 months after the end of an intervention [56–58]. Moreover, tDCS is easy to apply and is less prone to side effects than TMS. Compared with TMS, the lower focality of tDCS might be tolerable if the primary aim is a general modulation in the overall excitability rather than a causal proof of structure-function relationships in specific brain regions. This makes tDCS an appealing tool for neurorehabilitation settings. Indeed, there is an increasing interest in the application of tDCS to augment brain function in “home use” settings [59].

A common assumption is that anodal tDCS increases the overall activity in a brain region while cathodal tDCS decreases it, which should in turn map onto the respective behavioural consequences (i.e., improvement versus disruption) [60]. Indeed, a large number of previous studies used tDCS to facilitate learning and consolidation in different motor and cognitive tasks in healthy subjects [61, 62]. In contrast to TMS, the tDCS intensity is usually not calibrated to the individual motor threshold but given at a constant intensity across subjects. However, a better understanding of the recruitment of different neuronal circuits by tDCS could substantially advance the application of individualized stimulation protocols to facilitate treatment in therapeutic settings [63]. In this context, it remains to be determined whether stimulation paradigms that successfully modulate cortical excitability in healthy participants are also optimal in the diseased brain.

#### 2.2.1. Online and Offline tDCS

Similar to TMS, tDCS can also be applied before a certain task (offline) or during task processing (online). Online tDCS may induce slight shifts in task performance when considering a whole session [43], although the effect is not sufficiently strong to efficiently disrupt task performance on the single-trial level. The physiological effects of online tDCS might differ from those mediating short and long-lasting after effects [46, 64, 65]. As argued above, the immediate tDCS effects are presumably mediated by membrane depolarization for anodal stimulation and membrane hyperpolarization for cathodal stimulation [41], while the after effects could be explained by long-term potentiation and long-term depression [42, 66, 67]. The effects of tDCS on task processing can be quantified with behavioural measures, neurophysiological parameters, or neuroimaging read-outs.

Two quantitative reviews challenged the overall reliability and efficacy of tDCS to modulate cognitive functions [68, 69]. However, some of their analyses were limited by the inclusion of studies with methodological variations and the overall small number of comparable studies included [70, 71] and should thus be interpreted with caution. Indeed, a reanalysis on the included tDCS studies in the language domain pointed towards significant and reliable effects of single session anodal tDCS in the healthy brain [71], while the effects of cathodal tDCS might be more variable [72, 73]. To draw strong conclusions on the reliability of different tDCS protocols in various cognitive domains, replication studies with similar experimental paradigms and stimulation parameters would be mandatory, which are rarely available to date [74, 75]. Such studies might inform future applications of various tDCS protocols in therapeutic settings.

### 2.3. Novel Transcranial Electrical Stimulation Techniques

Recently, two novel NIBS techniques have been introduced: transcranial alternating current stimulation (tACS) [76] and transcranial random noise stimulation (tRNS) [77]. In contrast to the tDCS induced alterations of spontaneous cortical activity, these approaches are presumed to modulate oscillations of cortical networks in a frequency specific (tACS) or random manner (tRNS) [78]. The ability to modulate cortical oscillations provides a causal link between neural oscillations and specific cognitive processes [79–81]. tACS is usually applied with sinusoidal currents, although other waveforms are also possible. Depending on the applied frequency, tACS can be used to synchronize or desynchronize cortical oscillations and induce plastic effects in the stimulated areas. Synchronization is expected if a single resonance frequency is applied while desynchronization should result from the application of several frequencies [79]. tACS is assumed to entrain ongoing brain oscillations if the applied frequency matches the ongoing oscillation frequency in the brain [82]. Similar to TMS and tDCS, the effects of tACS can be quantified on the electrophysiological, neural, or behavioural level. To assess stimulation induced entrainment of brain oscillations, tACS can be combined with EEG or MEG. Since tACS induces strong artefacts in the EEG data, most of the previous studies relied on an offline approach. Only very recently, first successful correction methods for tACS induced artefacts in online EEG recordings were introduced [83]. These approaches might help to increase the current knowledge of the task-specific functional relevance of oscillatory brain activity. A different strategy was used by Lustenberger and colleagues [84] who introduced an EEG-feedback-controlled approach for real-time modulation of transient brain oscillations with tACS during sleep. Epochs of tACS in the spindle frequency range were triggered by the detection of sleep spindles with the analysis of EEG recordings being restricted to stimulation free intervals after short tACS epochs. Feedback-controlled tACS caused an enhancement of cortical synchronization in the spindle frequency range that intensified the spindling process and improved motor memory consolidation. These results demonstrate the value of oscillation-triggered stimulation to boost cognitive processes.

Other previous studies showed that tACS applied at individual alpha frequencies could enhance alpha band power in the subsequent offline EEG [85], with the after effects lasting for up to 30 minutes after the end of the stimulation [86]. As with other NIBS techniques, stimulation intensity might have an important influence on the effects of the tACS protocol. For instance, Moliadze and colleagues [87] demonstrated a nonlinear dependency between the intensity of their tACS protocol and the observed effects on motor excitability. Specifically, low stimulation intensities given at 140 Hz over the primary motor cortex resulted in cortical inhibition, as assessed with increased motor thresholds during simultaneous recordings of motor evoked potentials with single pulse TMS. In contrast, high intensities facilitated cortical excitability and decreased motor cortical thresholds. Interestingly, there were no significant effects for intermediate intensities, presumably indicating that inhibitory and excitatory effects cancelled each other out at such intensities. How this translates to areas outside the primary motor cortex and cognitive functions remains to be determined.

Recent studies reported sustained beneficial after effects when tACS was applied over left frontal cortex during explicit word pair encoding [88] or working memory processing [89] or over left temporoparietal cortex during implicit associative language learning [90]. In the latter study, 6 Hz tACS during language learning significantly improved retrieval performance in a collective of healthy young and older participants. The beneficial tACS after effect was driven by superior performance of older subjects after effective versus sham tACS, providing the first evidence that tACS might enhance language learning in the aging brain [90]. These results are encouraging with respect to a possible application of tACS in neurorehabilitation settings in the future.

tDCS can also be combined with tACS, with the alternating current being superimposed onto a direct current. This technique is referred to as oscillatory tDCS (otDCS) and aims at directly modulating the ongoing rhythmic brain activity at the frequency of the applied current [80, 81]. A number of studies used otDCS to modulate memory encoding or consolidation during sleep or wakefulness in the healthy brain [91–93]. For instance, Marshall et al. [91] applied anodal otDCS at 0.75 Hz after associative word learning to boost slow oscillations during sleep. This study showed a stimulation-induced increase in endogenous slow oscillatory activity and enhanced spindle activity. otDCS also improved declarative memory performance after sleep, demonstrating a causal role of slow oscillations in declarative memory consolidation. More recently, Ladenbauer and colleagues [94] found that otDCS enhanced slow oscillatory activity as well as fast spindle activity in older participants when applied during an afternoon nap. Moreover, otDCS improved picture memory retention after sleep. The authors concluded that otDCS during daytime naps might be used to counteract cognitive decline in aging.

As implicated by its name, transcranial* random noise* stimulation (tRNS) is applied with a broad frequency spectrum (0.1–640 Hz) and a random noise distribution [77] to cover physiological brain oscillations. On the physiological level, it is assumed that tRNS might induce LTP-like cortical plasticity by augmenting the activity of neuronal sodium channels in the stimulated parts of the brain [78]. Research on tRNS is still in its infancy, but some studies demonstrated that this technique might have lasting facilitatory after effects on motor cortical excitability. Accordingly, increases in the baseline levels of cortical excitability were shown to outlast the stimulation for up to 60 minutes when 10 minutes of high-frequency (100–640 Hz) tRNS was applied to the primary motor cortex [77]. Plastic after effects of tRNS on corticospinal excitability were already reported after a minimal stimulation duration of 5 minutes, but the respective after effects lasted for only 10 minutes [95]. Preliminary evidence in the study of cognition further suggests that tRNS might facilitate perceptual learning when applied over the visual cortex [96] but might disrupt categorical learning when given over the right dorsal lateral prefrontal cortex [78], with (unknown) task specific effects being likely to contribute to the direction of the tRNS induced effects.

In summary, most of the previous studies applied tACS and tRNS in the motor, visual, or auditory system to directly modulate cortical rhythms, but some studies also reported modulation of higher cognitive functions after tACS and otDCS (reviewed in [79–81]) or, more recently, tRNS [97, 98].

## 3. Combining NIBS with Neuroimaging Techniques

**Figure 1:**
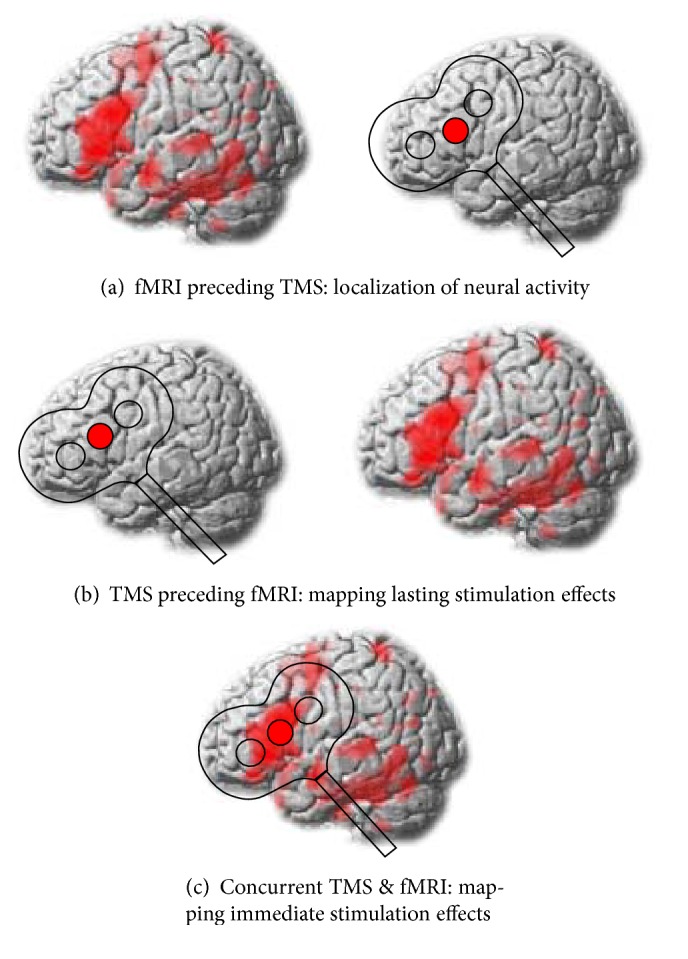
Illustration of different combinations of transcranial magnetic stimulation (TMS) and functional magnetic resonance imaging (fMRI). (a) fMRI can be used to localize target areas for TMS application. Subsequently, TMS is applied to probe the contribution of these regions to a specific task. (b) TMS can also be applied prior to fMRI to probe its lasting neuromodulatory effects on the network level. (c) Simultaneous TMS and fMRI can be used to map the immediate consequences of TMS on brain functions.

NIBS can be combined with functional neuroimaging to draw causal conclusions regarding the contribution of one or more specific brain regions to a given task or map NIBS induced changes on task-related activity and connectivity. An exemplified illustration of different combinations of TMS and fMRI is given in Figure 1. These combinations can also be used with tDCS, tACS, and tRNS. In a similar vein, the effects of NIBS can also be mapped with EEG or MEG, with the exception that the simultaneous combination of TMS and MEG is technically not feasible.

fMRI can be used prior to TMS to localize task-specific activity of interest and inform the subsequent application of TMS with respect to the stimulation site (Figure 1(a)). With this approach, TMS is used to probe the causal relevance of task-related activity observed with correlative neuroimaging measures. This approach has been used in most of the previous TMS studies in the language domain (for recent reviews see [26, 99]). A potential shortcoming of NIBS studies that solely focus on behavioural outcomes is that network effects can hardly be quantified unless multifocal NIBS is used. Moreover, the effects of NIBS might not necessarily map on the behavioural level and the contribution of remote effects in distant connected regions remains unclear. Although a common assumption of NIBS studies is that the strongest modulatory effect should occur at the targeted area, this might not necessarily hold true, especially for complex cognitive functions that depend on interactions of larger networks [22]. Indeed, remote effects outside the stimulated region have been reported to arise in neighbouring cortical regions close to the targeted area and in distant cortical and subcortical areas via intra- or interhemispheric connections [100, 101]. These effects are well described in the motor system [100, 102, 103], although it is less clear whether physiological remote effects are able to interfere with behaviour [104].

Plastic stimulation-induced changes on the network level can be mapped with fMRI after TMS application (Figure 1(b)). For instance, a recent study in healthy volunteers revealed an improvement in the subjects' ability to control impulsive responses after rTMS had been applied to the presupplementary motor area [105]. This beneficial after effect was mediated by increased activation and connectivity of a cortico-subcortical network including right inferior frontal gyrus and subthalamic nucleus. These results illustrate that the behavioural consequences observed with NIBS over a certain area might not necessarily be mediated by the stimulated area itself, but by spatially remote areas, which are part of the same network. Here, the combination of NIBS and neuroimaging or electrophysiological measures is well suited to map subtle changes in neural responsivity on the network level. In this context, it should be noted that TMS can also be given simultaneously during fMRI to probe the direct, immediate effects of the stimulation on the neural and behavioural level (Figure 1(c)). However, this combination is technically challenging and only established in a few research centres so far. The author is not aware of any concurrent TMS-fMRI application in the study of language. For a comprehensive overview of previous simultaneous TMS-fMRI studies, the reader is thus referred to a recent review [106].

## 4. Mapping Adaptive Plasticity in the Healthy Language Network

To date, only a few studies in the language domain combined NIBS with neuroimaging (i.e., PET or fMRI) or electrophysiological approaches (i.e., EEG or MEG) to map NIBS induced changes on the network level. These studies will be discussed in the next sections.

### 4.1. Evidence from Transcranial Magnetic Stimulation

One of the earliest studies that used TMS and PET to investigate speech perception in the healthy motor system was conducted by Watkins and Paus [107]. These authors found increased excitability of the primary motor cortex (M1) lip areas as measured by increased amplitudes of the motor evoked potentials recorded from orbicularis oris muscle during speech listening. Interestingly, increased motor excitability was positively correlated with an increase in the regional cerebral blood flow in left posterior inferior frontal gyrus (pIFG) during speech listening, indicating that the excitability of the M1 lip representation is influenced by input from the pIFG during speech perception. Additionally, increased cerebral blood flow in the left temporoparietal junction, an area previously associated with audio-motor-mapping processes during speech production [108], was also positively correlated with increased M1 excitability during speech perception [107]. The increase in motor excitability of the speech production system could reflect covert imitation or internal speech that might improve comprehension of the percept [99, 109]. The notion of a causal contribution of left M1 to speech perception was supported by several other TMS studies [35, 110].

More recently, Möttönen et al. [111] combined TMS and EEG to investigate how TMS modulates mismatch negativity (MMN) responses to phonetic changes in auditory vowels during automatic speech discrimination. In that study, subjects received 15 minutes of low-frequency 0.6 Hz rTMS over M1. Afterwards, participants listened to oddball sequences with frequent (“da”) and infrequent (“ba” and “ga”) phoneme stimuli while watching silent movies. The authors reported decreased MMN amplitudes to infrequent phonemes after disruption of M1 lip area but not M1 hand area. Moreover, the disruptive effect of rTMS was functionally specific since disruption of M1 lip area did not change MMN responses to nonverbal piano tones. These results provide further evidence for a causal contribution of the M1 lip area to speech discrimination.

In a follow-up investigation [112] MEG was used to track TMS-induced changes in the dynamic interaction between auditory and articulatory motor cortices during processing of attended versus unattended speech sounds. Again, subjects received 15 minutes of low-frequency rTMS over left M1 lip area. This study revealed a strong influence of attention on auditory-motor interactions. The authors found that TMS induced disruption of the motor lip representation modulated early, left-lateralized articulatory-specific responses in the auditory cortex that occurred 60–100 ms after sound onset when lip-articulated speech sounds were attended. In contrast, when speech sounds were ignored, rTMS disruption of M1 lip area led to late, nonspecific bilateral responses in the auditory cortices that started 170 ms after stimulus onset. These results show that the articulatory motor cortex contributes to the auditory processing of speech sounds and that attention can facilitate the interaction between auditory cortex and articulatory representations during speech perception.

In summary, these studies support the critical role of articulatory-motor representations in speech perception, a central notion of the motor theory of speech perception [113]. Moreover, these results provide novel insight into the interaction between articulatory-motor regions and primary auditory as well as language specific regions during speech perception.

TMS has also been combined with fMRI to investigate adaptive plasticity during speech production. For instance, a recent study investigated the contribution of the right hemisphere to speech repetition after focal disruption of the left hemisphere in healthy volunteers [114]. In that study, effective or sham continuous theta-burst stimulation (cTBS) was given over either anterior or posterior inferior frontal gyrus (a/pIFG) prior to neuroimaging in three separate sessions. Subsequently, participants had to overtly repeat real words and pseudowords (letter strings without any meaning) during functional MRI. Compared with sham cTBS or cTBS of neighbouring aIFG, cTBS of pIFG resulted in a strong suppression of task-related activity in the stimulated area and a strong upregulation of the contralateral homologous area during pseudoword repetition. Additionally, dynamic causal modelling (DCM) of functional MRI data was employed to investigate how TMS influences task-specific changes in the effective connectivity between homologous regions in the IFG. One important feature of DCM is that it provides a measure of both the strength and direction of neuronal interactions between prespecified regions of interest [115, 116]. Accordingly, effective connectivity analyses showed that right pIFG increased its facilitatory influence on left pIFG after left pIFG had been perturbed with cTBS. Critically, response speed became faster as the influence of the right pIFG on left pIFG increased, indicating that homologous areas in the right hemisphere can actively contribute to speech production after a focal left-hemispheric perturbation. These findings are compatible with the notion that increased activation of homologous right hemisphere areas might support aphasia recovery after left hemisphere damage (e.g., [117]; see discussion below).

In another recent study, Shinshi and colleagues [118] used TMS and MEG to investigate the role of the left pIFG in picture naming. In a first experiment, the authors showed that high-frequency 40 Hz triple pulse TMS over the left pIFG but not right pIFG significantly delayed naming latencies when applied 300 or 375 ms after picture presentation. The authors reasoned that TMS most likely disturbed the processes of syllabification during picture naming. To further elucidate the time course of picture naming, participants performed the same task during MEG. Interestingly, the authors found a significant correlation between the individual time period where TMS delayed picture naming and the individual time period when low gamma event-related desynchronizations peaked in left IFG, providing evidence for a critical contribution of these oscillations to picture naming.

Although the simultaneous application of TMS and MEG is technically not feasible since the TMS coil does not fit into the MEG helmet and TMS pulses might probably destroy the MEG sensors [119], another study successfully applied cTBS before MEG to modulate occipitoparietal alpha and gamma power during visuospatial attention processing [120]. In that study, offline inhibition of the frontal eye fields induced by cTBS caused a disruption of the attentional modulation of occipitoparietal alpha oscillations contralateral to the stimulated frontal eye field. This effect was explained by compensatory reorganization mechanisms in the dorsal frontoparietal attention network [120]. Such an approach would be of great interest to probe how inhibitory TMS over pIFG modulates neural oscillations during picture naming. This might provide new insight into the interactions and temporal dynamics between pIFG and other critical regions for picture naming such as posterior superior temporal gyrus.

TMS was also combined with neuroimaging and electrophysiological techniques to investigate language comprehension. For instance, in a number of studies, Andoh and colleagues investigated interhemispheric interactions during word recognition and auditory processing and at rest with consecutive TMS and fMRI [121–123]. In a first study, Andoh and Paus [122] combined high-frequency 10 Hz offline rTMS over left or right posterior superior temporal gyrus (pSTG) with subsequent fMRI to investigate auditory word comprehension. During fMRI, participants performed a word recognition task on native and foreign words. On the neural level, rTMS over either hemisphere resulted in an increase in the task-related activity in the nonstimulated homologous region. These changes were taken to reflect adaptive plasticity that compensated for the rTMS induced disruption of the respective other hemisphere. TMS-induced changes in task-related activity were accompanied by more specific modulations on the behavioural level. Hence, rTMS over left but not right pSTG selectively decreased response speed for native relative to foreign words. These results support the role of the left pSTG in lexical processing. However, it remains unclear whether the reported behavioural improvement was related to increased task-related activity in the contralateral right hemisphere or decreased activity at the site of stimulation in left pSTG [122]. In this context, future modelling studies might explore how changes in behavioural measures match TMS-induced modulations of effective connectivity measures between homologue regions.

In another study, Andoh and Zatorre [123] mapped TMS-induced modulations of interhemispheric interactions between auditory cortices with fMRI. Subjects performed a melody discrimination task during fMRI before and after cTBS over the right auditory cortex (AC). The authors reported increased task-related activity in the contralateral left AC after cTBS over right AC. The strength of the individual task-related upregulation of left AC was negatively correlated with behavioural performance. Hence, individual response speed decreased as activity in left AC increased and individuals with reduced contralateral activity did not exhibit any behavioural facilitation. Additionally, stronger interhemispheric connectivity between auditory cortices before cTBS was associated with faster response speed after cTBS. These results show how TMS modulates plastic short-term reorganization in the healthy auditory network. Similar mechanism might be observed after focal perturbation in the language system.

More recently, the same authors showed that rTMS over the right AC induces changes in functional connectivity in auditory and motor-related networks at rest [121]. To this end, healthy participants underwent resting-state fMRI prior to and after cTBS application over right and left AC and a control site in the vertex. The authors reported widespread changes in the functional connectivity in auditory and motor networks after cTBS of right AC in comparison to left AC. These network effects were underpinned by differences in the callosal tract integrity of auditory fibers, as evidenced by a negative correlation between the volume of the callosal auditory fibers and individual differences in the degree of cTBS-induced changes in functional connectivity between the auditory cortices. The authors concluded that their results support a role of the corpus callosum in mediating functional asymmetry. Together, their results emphasize the value of combining TMS and neuroimaging to map network effects of focal perturbations and investigate rapid short-term reorganization in auditory and language networks.

To map compensatory reorganization in the semantic system, two recent studies combined focal perturbation of the left anterior temporal lobe (ATL) with subsequent fMRI [124, 125]. In the first study, Binney and Lambon Ralph [124] found that cTBS over the left lateral ATL suppressed task-related semantic activity not only at the stimulated site, but also in other left-hemispheric areas of the semantic network, including the ventral ATL and ventrolateral prefrontal and posterolateral temporal cortex. Moreover, ATL suppression led to an extended, compensatory upregulation of the contralateral homologous region, indicating a high degree of adaptive plasticity in the semantic network. Congruent with the reported flexible adaptation of the semantic network, the second study from the same group [125] also found decreased activity in the left ventrolateral ATL after cTBS induced suppression of this region (relative to a control site in the occipital pole) and compensatory upregulation of the contralateral homologue. The upregulation of the right ATL was negatively correlated with task speed, indicating that subjects with shorter response latencies showed stronger right ATL activation. Additionally, effective connectivity analysis revealed that, after cTBS, the right ATL increased its intrinsic facilitatory influence on left ATL, demonstrating a flexible, bilateral organization of the semantic system with a strong degree of adaptive plasticity. In a behavioural experiment, cTBS also delayed task performance during synonym judgements, providing evidence for the functional relevance of this area for semantic processes. Together with the above-discussed study on pseudoword repetition [114], these results unravelled the compensatory potential of the right hemisphere during language production and comprehension and indicate a flexible, TMS-induced redistribution of the functional weight within a network for a specific language function.

**Figure 2:**
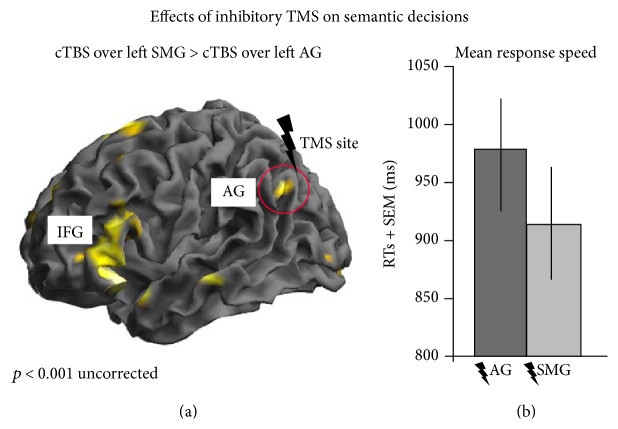
TMS-induced suppression of the semantic network during a word decision task in a representative subject. (a) Illustration of the strong remote effects induced by continuous theta burst stimulation (cTBS) given over the left angular gyrus (AG) prior to fMRI. Relative to cTBS over the neighboring supramarginal gyrus (SMG), cTBS of AG inhibited task-related neural activity during semantic decisions in the stimulated area as well as in the left inferior frontal gyrus (IFG) and in temporal regions. (b) Effects of cTBS on the mean reaction times (RTs) of semantic decisions. SEM = standard error of the mean.

Recently, we combined TMS and fMRI to investigate adaptive plasticity in the semantic network after focal perturbation of a key semantic area prior to task processing (Hartwigsen et al., unpublished data). That study revealed strong remote effects induced by cTBS of left angular gyrus (AG). Hence, cTBS suppressed neural activity not only at the stimulated site but also in remote semantic network areas, including left anterior inferior frontal gyrus and posterior middle temporal gyrus.  Figure 2 provides an illustration of these effects in a representative participant. Note that this participant also showed a strong delay in the semantic response speed after perturbation of the left AG. This effect was not significant at the group level, which might be explained by a strong compensatory upregulation of neighbouring parietofrontal regions for phonological processing (i.e., left supramarginal gyrus and pIFG) across participants. These findings implicate that the effects of TMS over a key area for a specific language function can modulate task-specific neural activity in the whole network. Moreover, the upregulation of neighbouring networks might help to maintain task function, indicating a high degree of flexibility in the language network to compensate for a focal perturbation of a key region.

One of the few studies that combined TMS and EEG in a consecutive fashion investigated the neural basis of semantic comprehension [126]. In that study, high-frequency 10 Hz rTMS was given in 500 ms trains over either left or right Wernicke's area (CP5 electrode in the 10-20 EEG system) or a control site in the occipital cortex 750 ms before stimulus onset during a picture-word verification task. To avoid stimulation-induced artefacts in the EEG signal, recordings of event-related potentials were stimulus-locked. The authors found a selective delay of response speed for artificial but not natural items with rTMS over left Wernicke's area. These effects were anatomically specific as rTMS over the right Wernicke homologue or occipital cortex did not have any disruptive effect. Moreover, on the electrophysiological level, TMS increased the amplitude of the late positive complex in the central-parietal electrodes of the right hemisphere. These changes were taken to reflect a compensatory transfer of language function from the left to the right hemisphere after disruption of the left hemisphere. The absence of any rTMS effect on natural items might indicate a more bilateral representation of sensory and perceptual features related to the processing of these items. These results show that adaptive plasticity and rapid short-term reorganization in the language network might also be mapped by changes in electrophysiological markers such as event-related potentials. Moreover, these results demonstrate a functionally relevant integration of right hemisphere activity into the normal language network subserving language comprehension on the word level [126].

In summary, the above cited studies stress the value of combining TMS with subsequent neuroimaging or electrophysiological techniques to map TMS induced changes in neural activity or oscillatory patterns and elucidate changes in inter- as well as intrahemispheric interactions after focal perturbation of task-specific key nodes.

### 4.2. Evidence from Transcranial Direct Current Stimulation

Very few studies combined tDCS with neuroimaging or electrophysiological techniques to investigate plastic changes in the healthy language network. Among these studies, Holland et al. [127] applied anodal tDCS over the left inferior frontal cortex (IFC) during concurrent fMRI to probe the role of the IFC in picture naming. Relative to sham tDCS, 2 mA of anodal tDCS significantly facilitated the response speed during picture naming. On the neural level, anodal tDCS significantly decreased task-related activity in inferior frontal regions, including the inferior frontal sulcus (IFS) and the ventral premotor cortex (PMv). Moreover, the observed individual behavioural facilitation was significantly correlated with the decrease in task-related activity in the stimulated left inferior frontal cortex. Although an association between a* decrease* in neural activity and behavioural* improvement* may sound counterintuitive at first glance, the authors argued that the underlying mechanism might be similar to neural priming effects reported in behavioural studies. Hence, anodal tDCS might have facilitated picture naming via regionally specific neural adaption in left inferior frontal cortex. Indeed, other studies also reported that beneficial behavioural effects of anodal tDCS were paralleled by a reduction in task-related activity in the left IFC ([128], see below for details).

In a recent follow-up investigation, Holland et al. [129] used dynamic causal modelling of functional MRI data to further elucidate tDCS-induced changes on task-related interactions in the left IFC during picture naming. In that study, the authors explored how anodal relative to sham tDCS changes task-related interactions between the left IFS and PMv. Results revealed that the previously observed significant decrease in task related activity of both frontal nodes with anodal tDCS was underpinned by an increase in the inhibitory feedback influence of IFS on PMv. These results presumably reflect neuronal adaption and more efficient task processing. Moreover, the individual variability in the feed-forward connection strength from PMv to IFS was positively correlated with the degree of facilitation in picture naming during anodal tDCS. According to the authors, their results indicate that anodal tDCS reduced noise in the naming system and thereby made the signal (i.e., the correct word related to a presented picture) easier to detect. This might indicate that the correct word was easier to select among the competing alternatives (the noise) in the mental lexicon with anodal tDCS. The authors further argued that anodal tDCS might have worked as a top-down mechanism that filtered out irrelevant signals by reducing “noisy” activity in left PMv. These results underline the important role of left IFS as top-down node and driver during speech processing. Moreover, these findings demonstrate the value of combining NIBS and modelling of fMRI data to provide insight into the interactions between task-specific regions in speech and language networks.

These results were complemented by a recent concurrent tDCS-fMRI study that probed the neural correlates of tDCS-induced facilitation over left IFG during word production [128]. In that study, 1 mA anodal or sham tDCS was applied in the MR scanner for 20 minutes. This included a resting state fMRI session and subsequent task-related fMRI with a semantic word generation paradigm. Relative to sham tDCS, anodal tDCS significantly improved semantic fluency by increasing the number of correctly produced responses for different visually presented categories. The beneficial behavioural effects were paralleled by a reduction in the task-related activity in the left ventral IFG. Functional connectivity analyses of resting-state fMRI data revealed increased coupling between the left IFG and other core areas for language processing, including left middle temporal gyrus and bilateral inferior frontal, inferior parietal, and prefrontal regions during resting-state fMRI. The authors suggested that tDCS modulated endogenous low-frequency oscillations in the language network that might have induced more efficient task processing in relevant network nodes and could thus explain the observed behavioural improvement. Accordingly, it was further speculated that the modulation of endogenous low-frequency oscillations was not restricted to the targeted area but also spread to functionally connected brain areas, which would explain the observed network effects. In this context, it would be of great interest to explore how tDCS influences the* task-related* effective connectivity between these regions. Moreover, a frequency specific modulation with tACS might further elucidate the functional relevance of endogenous oscillations.

The electrophysiological underpinnings of tDCS-induced facilitation on language production were investigated with combined tDCS and EEG by Wirth et al. [130]. To provide a comprehensive characterization of the effects of tDCS on event-related potentials as well as neural oscillations and behavioural parameters, the authors explored both direct (i.e., online) and after (i.e., offline) effects of tDCS. To this end, 1.5 mA of anodal or sham tDCS was applied for 30 minutes over the left dorsal prefrontal cortex. The task consisted of a semantic interference paradigm that prompted subjects to name repeatedly presented pictures of objects displayed in semantically homogenous (e.g., different fruits) or heterogeneous contexts (e.g., a fruit and an insect), with the presence of categorical similar objects inducing lexical-semantic competition. Anodal tDCS significantly reduced the semantic interference effect for homogenous contexts and thus facilitated picture naming latencies. In contrast, on the electrophysiological level, this effect was underpinned by an enhanced semantic interference effect (i.e., increased event-related potentials) for left but not right temporal electrode sites. These results were taken to reflect a superior tuning of neural responses with language-related generators. Specifically, the authors suggest that the behavioural tDCS effect might be related to increased prefrontal inhibitory functions, reflecting increased processing efficiency. In contrast, the electrophysiological effect might have resulted from a network effect in the temporally distributed representational system. With respect to the after effects of anodal tDCS, the authors reported a significant reduction in delta activity at rest and during picture naming after offline tDCS over dorsal prefrontal cortex. Since activity in the slow-wave delta band might be regarded as a surrogate of neural inhibition [131], these effects were interpreted as neural excitation (i.e., disinhibition) and suggested to reflect a boost in neurocomputational resources. Future studies should explore whether the behavioural results are directly related to the observed electrophysiological changes. In this context, different tasks might be related to different oscillation frequencies that could be selectively modulated by tACS protocols.

The author is not aware of any previous study in the language domain that combined cathodal tDCS with fMRI or EEG to investigate the neural correlates of inhibitory stimulation. Only a few studies used cathodal tDCS to probe the effects of LTD-like plasticity on language learning. One of these studies compared the effects of repeated 20 minutes sessions of cathodal, anodal, or sham offline tDCS over the left primary motor cortex prior to action word learning over four consecutive days [132]. The action word learning paradigm included correct and incorrect couplings of pictures of concrete body-related actions (e.g.,* shaving*) with meaningless pseudowords (e.g.,* apef*). Correct couplings were more frequent than incorrect ones and subjects had to indicate via button press whether picture and word matched. The authors found that the number of novel action words successfully translated into German at the end of training was significantly reduced after cathodal versus sham tDCS. In contrast, anodal tDCS did not significantly affect task performance. This effect was anatomically specific, as tDCS over the left dorsolateral prefrontal cortex did not affect translations after language training. The effect was also functionally specific since tDCS did not disrupt non-action related object word learning in a control experiment. Additional analyses further explored the nature of the disruptive effect of cathodal tDCS: Relative to sham stimulation, cathodal tDCS significantly reduced success rates in vocabulary acquisition. Specifically, cathodal tDCS decreased the ability to associatively couple actions with novel words (i.e., to identify correct couplings), providing evidence for a causal involvement of left primary motor cortex in the acquisition of novel action-related words. The authors argued that the process of correct couplings between words and actions might strongly rely on synaptic strengthening between motor and language areas and might thus be particularly susceptible to a stimulation-induced downregulation of cortical excitability. Future studies should explore the network effects of cathodal tDCS on language learning with combined tDCS and neuroimaging. In particular, if the explanation of a task-specific synaptic strengthening was true, one might expect that cathodal tDCS over the primary motor cortex affects the interaction between motor and inferior frontal language regions during language learning.

Although the precise physiological mechanisms of the tDCS-induced modulation of (language related) neural activity remain unclear, the above described studies that combined tDCS with concurrent neuroimaging [127, 129, 133] implicate that the beneficial effects of anodal tDCS on different language functions might be explained by an increase in the efficiency of task processing locally in the stimulated areas as well as in interconnected language regions. It remains to be determined how anodal and cathodal tDCS affect other language functions.

## 5. Implications for Aphasia Recovery after Stroke

The above discussed studies demonstrate the value of combining noninvasive brain stimulation with electrophysiological and neuroimaging measures to map NIBS induced changes on the network level. Although the overall number of multimodal studies in the language system is scarce, first results implicate that a focal perturbation of a strategic language region changes the functional weight within in a network, which may result in a compensatory upregulation of neighbouring left-hemispheric regions or contralateral right-hemispheric regions. Moreover, NIBS induced changes on the behavioural level might be mediated via modulation of the interaction and effective connectivity within a network for a specific language function. These results provide important insight into the compensatory potential and flexible redistribution of the human language system and might be transferred to the lesion brain to increase the current knowledge of the dynamics of reorganization in the language network after brain lesions.

### 5.1. Current NIBS Approaches to Support Language Recovery after Stroke

Indeed, an increasing (yet still relatively small) number of studies have used NIBS to promote language recovery in poststroke aphasia (for recent reviews see [134, 135]). Most of these studies relied on low-frequency rTMS to suppress language-related activity in the “overactive” right IFG (see [136, 137]). More recently, language therapy was also combined with tDCS, as this technique is cheap and easy to apply and less prone risk to severe side effects than TMS. While the results from these studies are generally encouraging, the reported effect sizes are not striking and the potential benefit of TMS and tDCS in the neurorehabilitation of language functions remains elusive.

A Cochrane review that included 6 tDCS studies and a total of 66 patients came to the conclusion that there is currently no evidence for the effectiveness of anodal or cathodal tDCS to improve language functions in poststroke aphasia [138]. However, these results should be interpreted with caution since they were obtained from heterogeneous studies that differed with respect to aphasia type and severity, as well as stimulation parameters [137]. Indeed, a recent meta-analyses including 6 inhibitory rTMS studies and 3 cathodal tDCS studies that aimed at inhibiting right-hemispheric regions in subacute or chronic patients with poststroke aphasia indicated positive effects of NIBS on naming accuracy [139].

It should be borne in mind that the use of NIBS to facilitate language recovery in patients with aphasia after stroke is still at its infancy and future studies with larger collectives are needed to provide a systematic investigation of the potential of different NIBS approaches to effectively modulate language functions across the time course of recovery.

To date, only very few studies investigated the neural correlates of NIBS induced modulation in the lesioned language network. For instance, a recent study by Heiss et al. [140] applied inhibitory rTMS over the contralesional right anterior IFG or vertex (control site) in a relatively large group of 29 right-handed and two left-handed aphasic patients in the subacute and chronic phase after left-hemispheric stroke. To this end, 10 sessions of 1 Hz effective or sham rTMS were combined with speech and language therapy. Concordant with other rTMS studies in patients with poststroke aphasia [141–145], the authors reported improvement of language functions after rTMS over the IFG but not vertex in right-handed patients. PET measurements revealed a shift of language-related activity towards the left hemisphere in treated right-handers, while the vertex group maintained activation in the contralesional hemisphere. Interestingly, language improvement was also found in the two left-handed patients although PET scans demonstrated only a very small interhemispheric shift and a consolidation of active networks in both hemispheres. These results indicate that rTMS-induced suppression of “maladaptive” right-hemispheric activity might be beneficial to facilitate language recovery after stroke, probably via reshifting language activity towards perilesional left-hemispheric regions. This would be compatible with the notion that, after left-hemispheric stroke, the right hemisphere is released from transcallosal inhibition and might in turn suppress (beneficial) language-related activity in perilesional regions [134]. The beneficial effects of rTMS over the contralesional right IFG to improve picture naming abilities in patients with chronic poststroke aphasia were replicated in other studies by the same group [143, 146]. Recently, a first multicentre study on the efficacy of inhibitory NIBS over the contralesional hemisphere to support aphasia recovery was launched (see [147]).

To account for the large individual variability in response to various stimulation protocols reported in the literature and optimize individual montage for treatment in 12 patients with chronic nonfluent aphasia after stroke, Shah-Basak et al. [148] investigated different tDCS set-ups. Despite individual variability, best improvement was on average obtained after 10 days of cathodal stimulation given over the left frontal cortex during picture naming. Moreover, improvement of aphasia severity lasted for at least 2 months after the intervention, indicating that repeated individualized tDCS treatment might have lasting effects on language recovery after stroke.

In contrast, the beneficial effect of perilesional* facilitation* was explored by Szaflarski and colleagues [33] who demonstrated improved semantic fluency in 8 patients with chronic poststroke aphasia after intermittent theta-burst stimulation over the left IFG. The beneficial effect of facilitatory stimulation was underpinned by a leftward shift in language-related activation during subsequent fMRI.

Congruent with the previously observed improvement after perilesional facilitation [33], a recent study showed that anodal stimulation of the left primary motor cortex improves language recovery in patients with chronic aphasia after left-hemispheric stroke [149]. In that study, picture naming therapy over 2 weeks was combined with anodal or sham tDCS in two groups of aphasic patients. The authors reported significant improvement directly after treatment in both groups, with a slightly larger effect for trained items and a significantly larger effect for untrained items in the anodal tDCS group. Importantly, in a 6-month follow-up, treatment effects were significantly larger in the anodal tDCS group and transfer effects were only maintained in these patients. Moreover, functional communication was also more improved with anodal tDCS at both time points. Together, these results highlight the beneficial contribution of motor regions (or functionally connected areas) to language recovery and indicate that anodal tDCS effects might generalize to measures of functional communication that are highly relevant for everyday life.

Recently, the combination of contralesional inhibition and perilesional facilitation was established. For instance, Khedr and colleagues [150] reported significant language improvements after 10 repeated sessions of dual-site TMS combining inhibitory rTMS over the right Broca homologue with subsequent facilitatory TMS over the affected left-hemispheric Broca region in 13 patients with subacute aphasia (compared with 7 patients receiving sham rTMS). The beneficial effects of effective relative to sham TMS remained significant for 2 months after the end of treatment. Future studies in larger collectives should explore whether dual-site TMS proves more effective than unilateral TMS.

### 5.2. Open Questions and Future Directions

The above cited studies suggest that the combination of NIBS and language therapy might be promising to support aphasia recovery after stroke. Future studies in larger collectives should explore whether individual lesion size, location, and symptoms could be used to predict the efficacy of a specific individualized NIBS approach (cf. [148]). Indeed, it was suggested that the strong individual variation in response to different tDCS protocols might reflect differences in neural recovery mechanisms [151]. Of note, most of the previous studies investigated the beneficial effects of NIBS to support aphasia recovery on the behavioral level only. To increase the currently limited knowledge of (individual) recovery mechanisms, future studies should also elucidate the neural underpinnings of these effects in the reorganized brain.

As a first step, the results from studies in the healthy language network should be transferred to the lesioned brain to explore how NIBS can modulate neural activity and connectivity in the reorganized language system. In this context, it is worth to bear in mind that cognitive processes are not mediated by isolated neural areas but rather engage dynamic interactions among relevant regions [152]. In particular, measures of functional and effective connectivity [153, 154] might capture NIBS induced changes in the causal network organization that might be more closely related to the neurobiological mechanisms by which NIBS changes a cognitive function compared to only analysing regional changes in neural activity [155]. Consequently, future patient studies should include the systematic application of both inhibitory and facilitatory NIBS protocols over perilesional as well as contralateral regions. Subsequent neuroimaging might elucidate mechanisms of plasticity in the reorganized language system. A better understanding of both the healthy and the lesioned brain's potential for adaptive plasticity would be mandatory to increase treatment efficiency in poststroke aphasia. Here, it is important to appreciate that recent tDCS results point towards altered functions of synaptic plasticity in the aging brain [156], which is of particular importance for the application of NIBS for neurorehabilitation purposes.

To provide further insights into the dynamics of network reorganization in the lesioned language network, longitudinal designs should explore the efficacy of different NIBS protocols across the time course of language recovery. This may include the application of different stimulation protocols during different phases of reorganization after stroke [157, 158]. These approaches should be informed by current models of language recovery after stroke. For instance, Saur et al. [117] argued that the contribution of perilesional and homologous right-hemispheric regions might change across the time course of recovery. Employing a longitudinal fMRI design, these authors reported that patients with poststroke aphasia after left-hemispheric stroke showed a global downregulation of language related activity in the acute phase after stroke. In the early subacute phase, language improvement was correlated with increased activity of the right hemisphere, with the strongest peak observed in the right Broca-homologue. In contrast, in the chronic phase, a normalization of language activity with a reshift towards the dominant left hemisphere was associated with further improvement. Although the role of the right hemisphere in language recovery after left-hemispheric stroke is still debated, this study implicates that an early, temporary recruitment of contralesional homologous regions may be beneficial, while longer-term language improvement is associated with a recruitment of perilesional left-hemispheric regions (see [159, 160]). Accordingly, Winhuisen et al. [161] argued that restoration of the left-hemispheric language network is more effective for language recovery after stroke, but in some cases, the right hemisphere is successfully integrated. Hence, the dynamic process of language recovery may involve a variety of plastic changes in both hemispheres [134]. With small left hemisphere lesions, complete or near-complete recovery may be achieved by recruitment of perilesional regions [162]. In contrast, for larger left hemisphere lesions, additional right hemisphere recruitment may subserve language functions, although such remodelled language networks might be less efficient than the premorbid left-hemispheric network [163]. Notably, additional factors such as premorbid laterality of language function and lesion site are important determinants of successful integration of right-hemispheric activity during poststroke reorganization in language networks [158].

A beneficial contribution of right hemisphere regions after left-hemispheric lesions further converges with the findings of the above discussed studies in healthy volunteers (cf.  Section 4.1) that TMS-induced perturbation of the left hemisphere induced a compensatory upregulation and contribution of the right hemisphere during speech repetition [114] and language comprehension [122, 124–126]. This might indicate that the right hemisphere has the potential to support language functions of the dominant left hemisphere, probably by contributing more domain-general or supralinguistic functions such as (emotional) prosody and perceptual features of word stimuli. Indeed, it was argued that, in general, engaging the contralateral homologous area helps to preserve behaviour by taking over the specific function of the left hemisphere or contributing coarser computations for the same general processes [164, 165]. Notably, the beneficial effects of an acute flexible integration of homologous right-hemispheric regions after a left-hemispheric lesion might be restricted to the initial stages of adaptive compensation. Indeed, the effects of acute short-term plasticity induced by rTMS in the healthy network are most comparable with the immediate reorganization effects in the acute phase after stroke [163].

**Figure 3:**
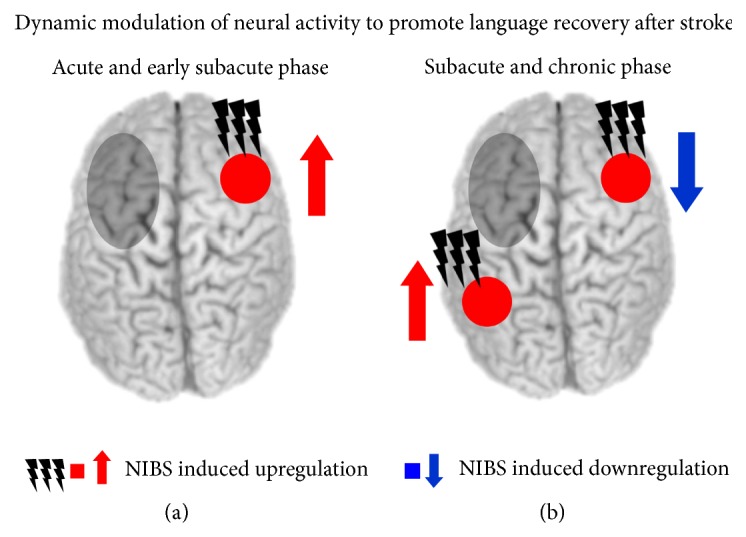
Illustration of a phase-specific stimulation approach to promote language recovery after left-hemispheric stroke. Note that the appropriate stimulation protocol might strongly depend on the site and size of the lesion and the individual deficits. (a) In the acute and early subacute phase, an upregulation of homologous right-hemispheric regions with facilitatory noninvasive brain stimulation (NIBS) might promote language recovery. (b) In the late subacute and chronic phase, patients might rather benefit from an inhibition of homologous right-hemispheric regions and an upregulation of ipsilesional regions by NIBS. Grey circles illustrate a stroke-induced lesion.

The above discussed results are summarized in a phase-specific NIBS approach to promote language recovery after left-hemispheric stroke in Figure 3. This model assumes that the contribution of left- and right-hemispheric regions to language recovery might change over time with an early beneficial contribution of right-hemispheric regions in the acute and early subacute phase after stroke and a stronger reshift of language-related activity to remaining left-hemispheric regions in later subacute and chronic phases.

It should be noted that although the majority of studies applied NIBS over the same regions across patients, irrespective of the individual lesion site and size, future studies might rely on individual recovery maps obtained from neuroimaging to identify target areas for NIBS across the time course of recovery. Most of the previous studies applied NIBS over the left or right anterior IFG to facilitate language recovery after stroke. However, at least with focal TMS, the effect might critically depend on the targeted subregion within the IFG (i.e., anterior versus posterior part) and the task under investigation. In this context, the systematic application of different NIBS protocols over various temporal regions should also be addressed. This should include the comparison of unifocal TMS effects with dual-site stimulation ([150], see above) and the systematic investigation of bilateral tDCS effects, for example, with the anode placed over a left-hemispheric language region and the cathode over the right-hemispheric homologue [166]. Other studies suggest that modelling the current flow on an individual basis might help to optimize NIBS effects at the target region and might thus be used to increase therapeutic efficiency in future studies [167].

Only recently, NIBS was combined with patient-relevant outcome measures such as improvement in functional communication ([149], see above). Such approaches are mandatory to assess the ecological validity of NIBS effects.

One remaining open question is related to the efficacy of novel modulatory techniques such as tACS and tRNS to modulate language-related neural oscillations and thereby facilitate aphasia recovery. For instance, language recovery was associated with a decrease in the perilesional delta power in previous studies [168, 169]. Hence, future studies might probe whether NIBS induced modulation of abnormal slow wave patterns might be beneficial.

Finally, future studies in both the healthy and lesioned brain are mandatory to shed light on the biological mechanisms of plastic changes induced by different NIBS protocols. Here, simultaneous combinations of NIBS and electrophysiological or neuroimaging methods as well as the combination of NIBS and modelling approaches are particularly promising.
